# Supplementary figures and images for: Genome-scale CRISPR screening reveals that C3aR signaling is critical for rapid capture of fungi by macrophages
Source: PLoS Pathog. 2022 Sep 29;18(9):e1010237. doi: 10.1371/journal.ppat.1010237 (PMC9578593; doi:10.1371/journal.ppat.1010237)

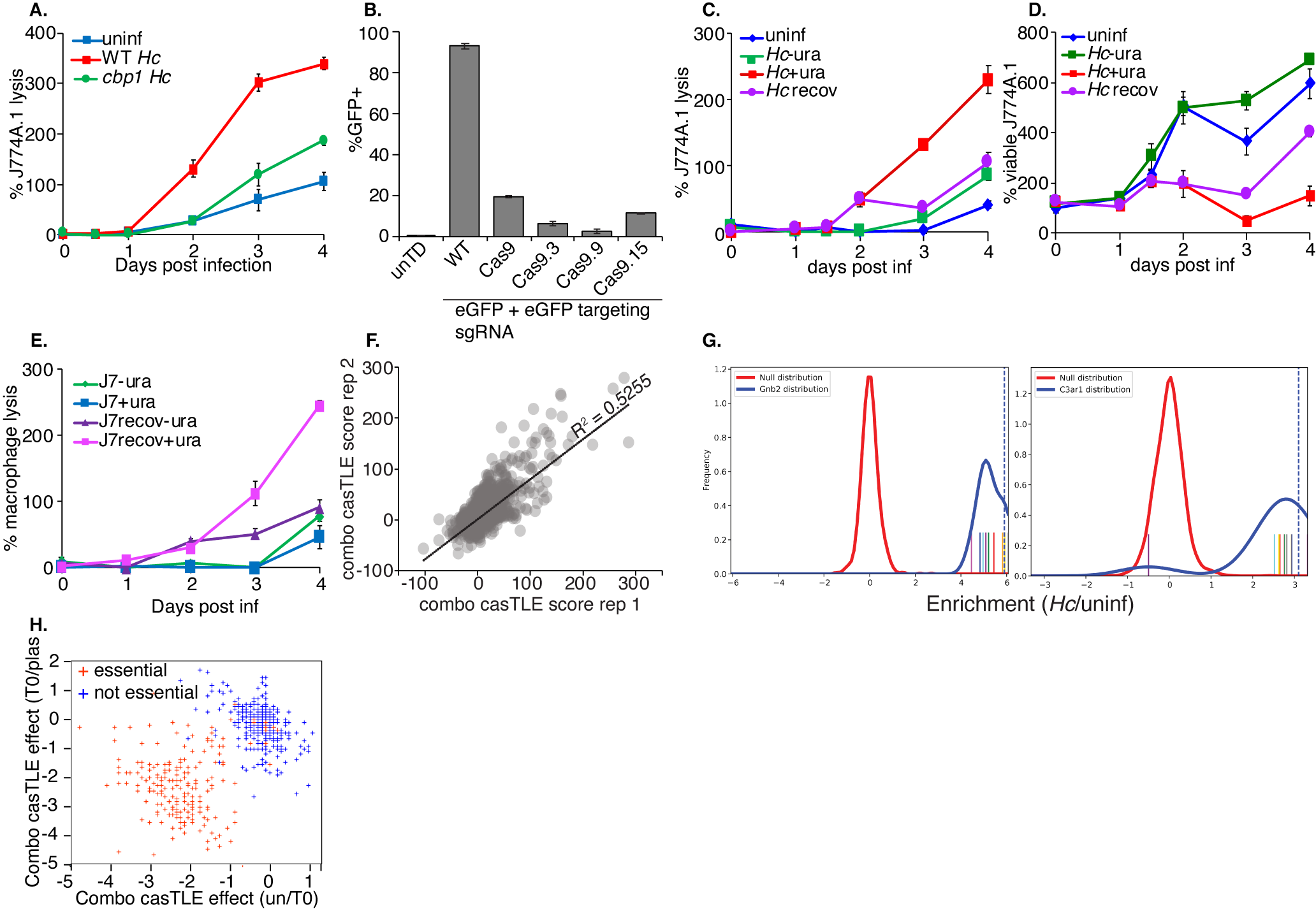

Supplement: S1 Fig — A. Characterization of Hc-mediated lysis in J774A.1 macrophage-like cells. J774A.1 cells were infected with WT Hc, or Hc with a disruption in a gene, CBP1, that is required for Hc to lyse macrophages. Lysis over time was measured using the LDH release assay. B. Validation and clonal expansion of Cas9-expressing J774A.1 cells. Cells were transduced with an Ef1a-Cas9-Blast expression vector and grown under blasticidin selection to generate a population of Cas9-expressing cells. These were subjected to single-cell sorting and clonal expansion to generate Cas9-expressing J774A.1 clones with high Cas9 activity. Cas9 activity was measured by transducing J774A.1 cells with a guide RNA vector that co-expressed EGFP with a sgRNA targeting EGFP. Cas9 activity leads to silencing of the GFP following puromycin selection. Cas9 clone 9 was chosen for the large-scale CRISPR screens due to its high-efficiency GFP silencing. C-D. Characterizing lysis and recovery from infection with uracil pulses during infection with a Ura5-deficient Hc. J774A.1 macrophages were infected with ura5 mutant Hc in the presence or absence of exogenous uracil (0.4ug/mL). Uracil-containing cells were washed and media was replaced with uracil-poor media after 2d of lysis, which allowed the macrophages to recover. Recovery was assessed using LDH release quantification to assess lysis, and the confluency of viable cells in the wells was estimated using the pico-green dsDNA assay kit following lysis of macrophages with water. E. Macrophages that had been recovered from lysis by removal of uracil from culture media were passaged for several days, and uracil was added to selected wells. Macrophage lysis over time was monitored by assessing LDH release over time to determine whether dormant yeast would be able to re-activate upon introduction of uracil. F. Reproducibility of the casTLE score across two replicates of the screens. G. Histograms comparing the distribution of negative control sgRNAs and sgRNAs targe [file ppat.1010237.s001.tif]

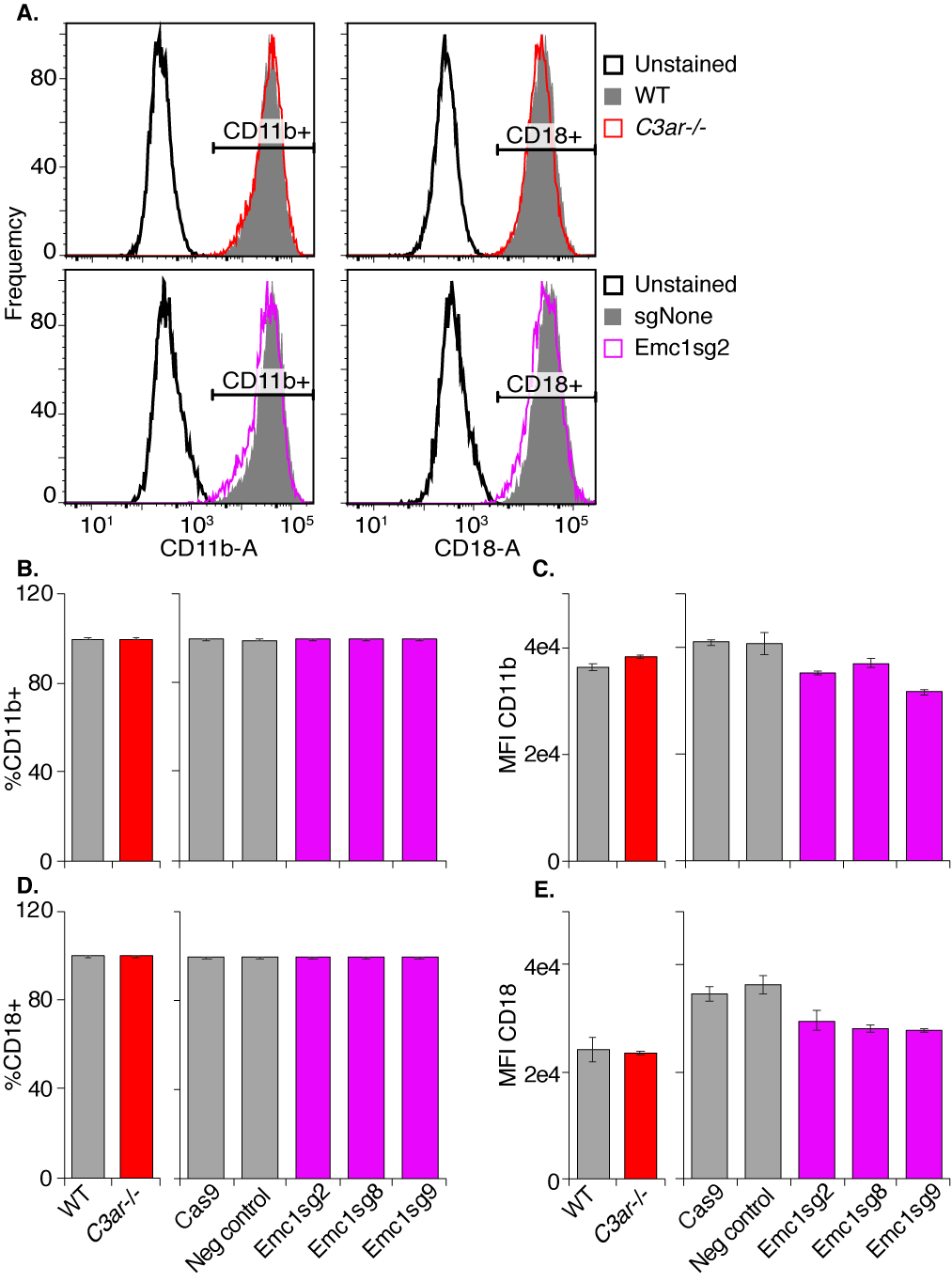

Supplement: S2 Fig — BMDMs from C3ar-/- and WT mice, in addition to BMDMs expressing Cas9 and control or Emc1-targeting sgRNAs, were stained with anti-CD18 and anti-CD11b antibodies and analyzed by flow cytometry (n = 2 biological replicates). A. Representative histograms showing CD11b and CD18 levels in control, C3ar-/-, and Emc1 CRISPRKO BMDMs. The percentage of CD11b (B) and CD18 (D) positive macrophages was analyzed. The mean fluorescence intensity of CD11b (C) and CD18 (D) were also measured. (TIF) [file ppat.1010237.s002.tif]

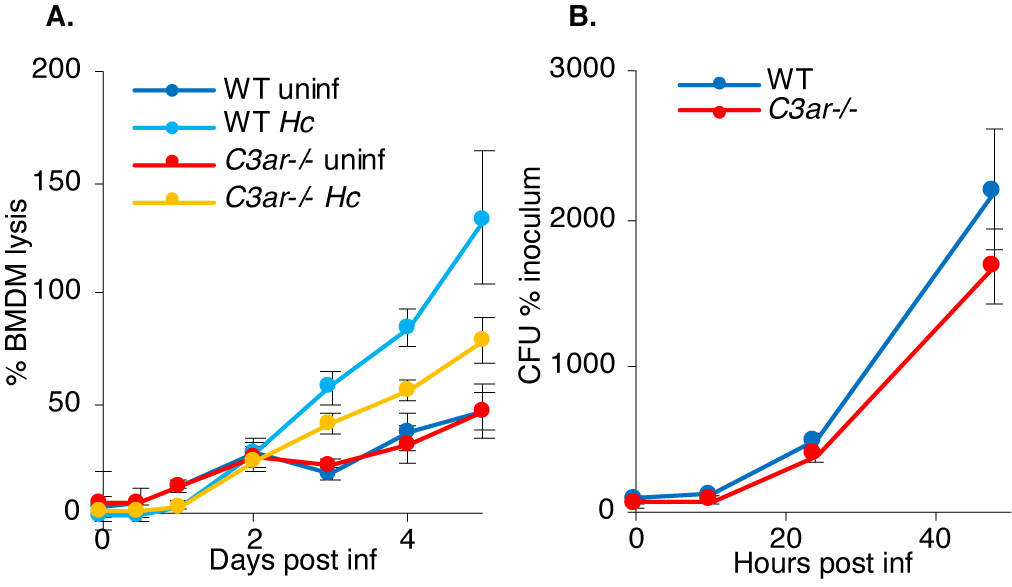

Supplement: S3 Fig — BMDMs were infected with Hc (MOI = 0.5), and macrophage lysis was quantified by measuring the release of lactate dehydrogenase (LDH) into the culture supernatants over-time (n = 3 biological replicates, 3 technical replicates/biorep) (A). LDH release is presented as the percentage of total LDH present in the well (supernatant and macrophage lysate) at 2 hours post-infection. At the indicated time points, macrophages were lysed using water, and lysates were spread on agar plates. Colony forming units (CFUs) were enumerated (n = 3 biological replicates, 2 technical reps/biorep) (B). (TIF) [file ppat.1010237.s003.tif]

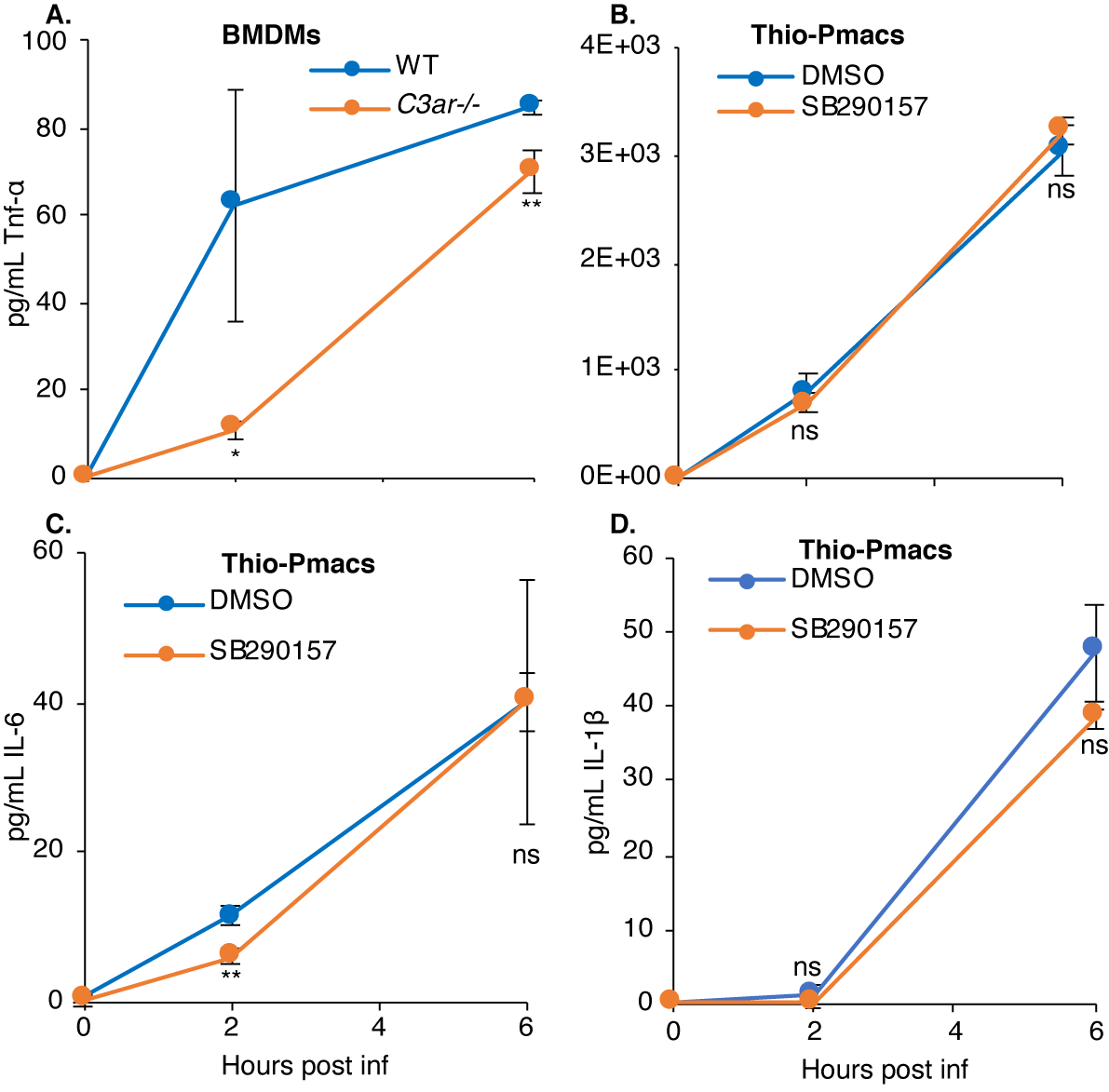

Supplement: S4 Fig — A. WT and C3ar-/- BMDMs were infected with Hc (MOI10), and TNFα levels in macrophage supernatants were measured using the BD Cytometric Bead Array (CBA) kit (n = 3 biological replicates). B-D. Thioglycollate-elicited peritoneal macrophages were treated with the C3aR antagonist (SB290157, 10 μM) and infected with Hc (MOI10). TNFα (B), IL-6 (C), and IL-1β (D) levels in culture supernatants were measured by CBA. *p ≤ 0.05, **p ≤ 0.01, ns = not significant by two-tailed student’s T-test. (TIF) [file ppat.1010237.s004.tif]

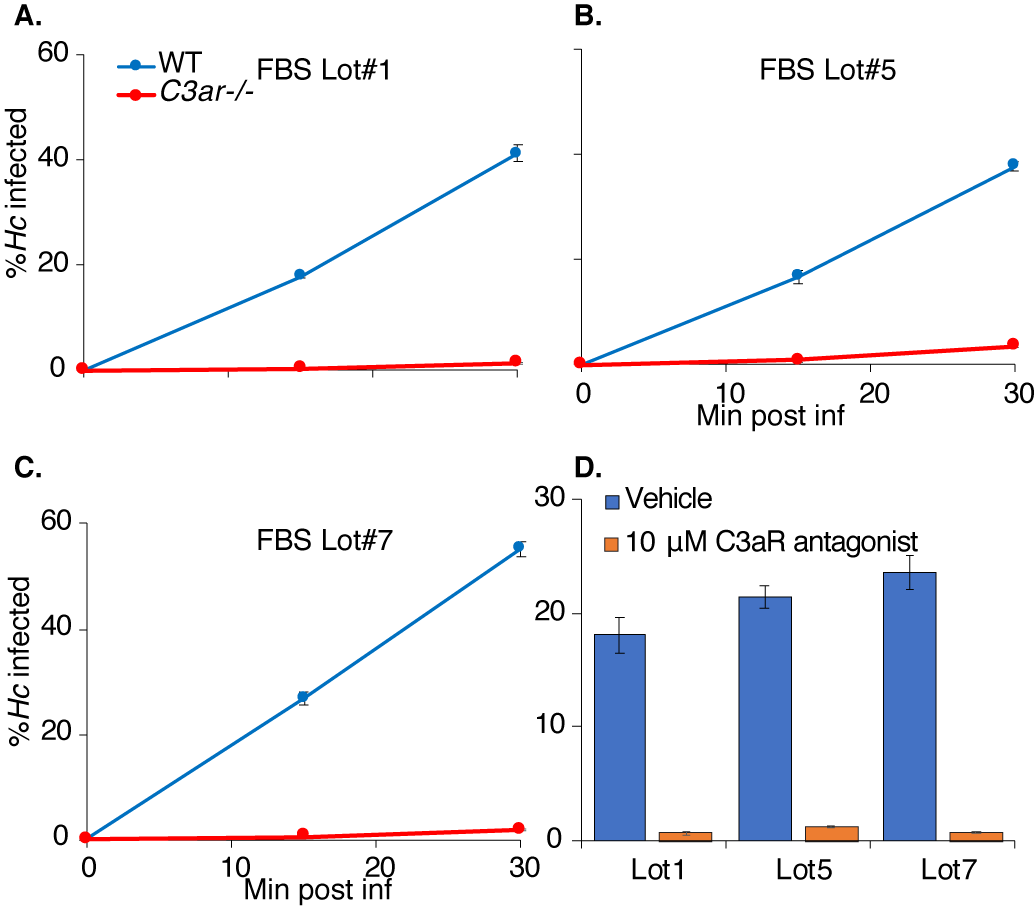

Supplement: S5 Fig — (A-C) BMDMs from WT and C3ar-/- mice were infected with Hc in the presence of 20% FBS from three different lots from 2 separate suppliers. In addition, WT BMDMs differentiated in different lots of serum were treated with 10 μM of the C3aR antagonist and infected with Hc (B). Phagocytosis of Hc was measured by flow cytometry as described previously (n = 2 biological replicates). (TIF) [file ppat.1010237.s005.tif]

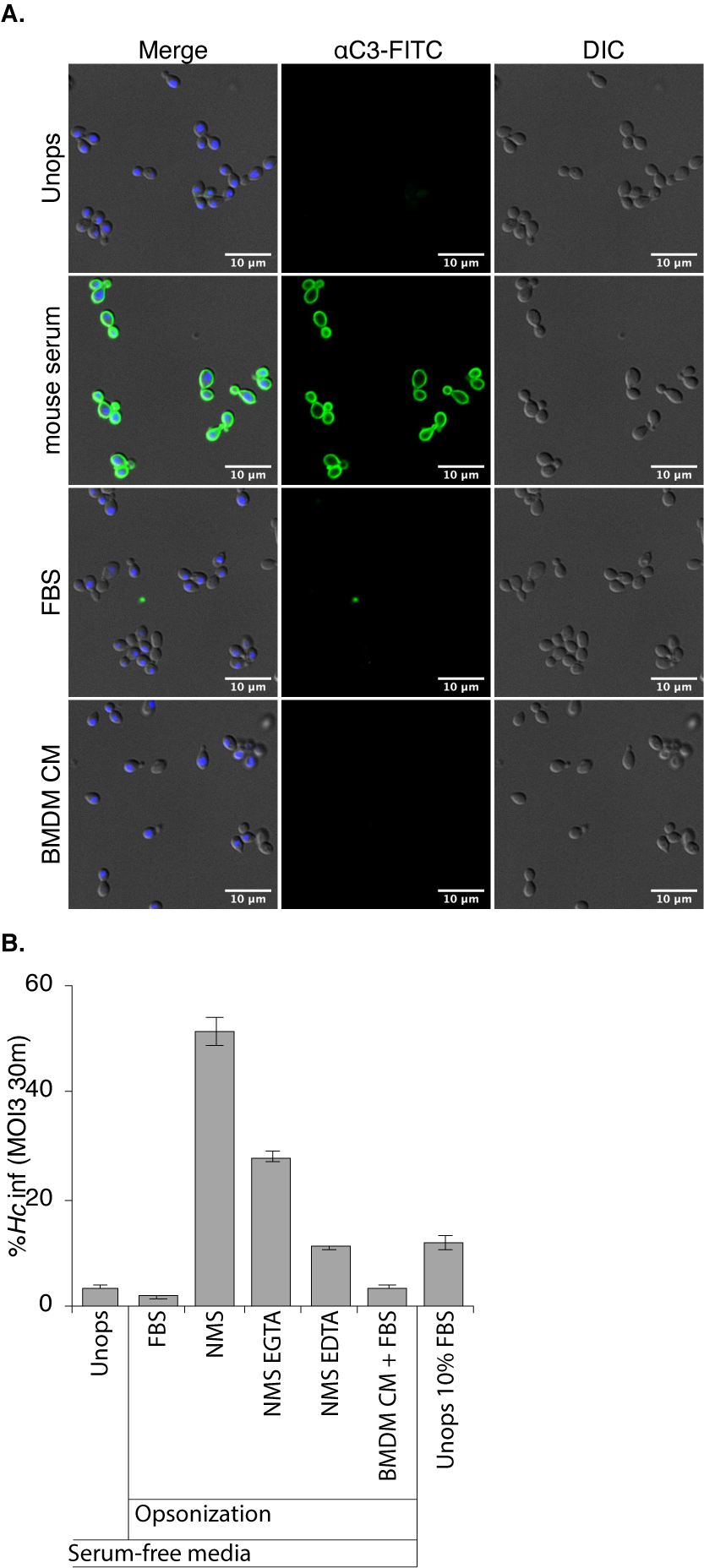

Supplement: S6 Fig — BMDMs were cultured in media containing 10% FBS, and the BMDM conditioned media was harvested. Hc was incubated with macrophage conditioned media (BMDM CM), 10% FBS, or 10% normal mouse serum (NMS) with 10 mM EGTA or EDTA as indicated for 30 min 37°C. A. Incubation with conditioned media or FBS does not lead to C3 deposition on the Hc surface. C3 deposition on Hc yeast was analyzed by immunofluorescence microscopy using an anti-C3 antibody. Scale bar = 10 μm. B. Pre-incubation of yeast with conditioned media does not improve macrophage phagocytosis of Hc. Yeast were washed and used to infect BMDMs (MOI3, 30 min) in serum-free media. Phagocytosis was assessed using flow cytometry as previously described (n = 3 biological replicates). (TIF) [file ppat.1010237.s006.tif]

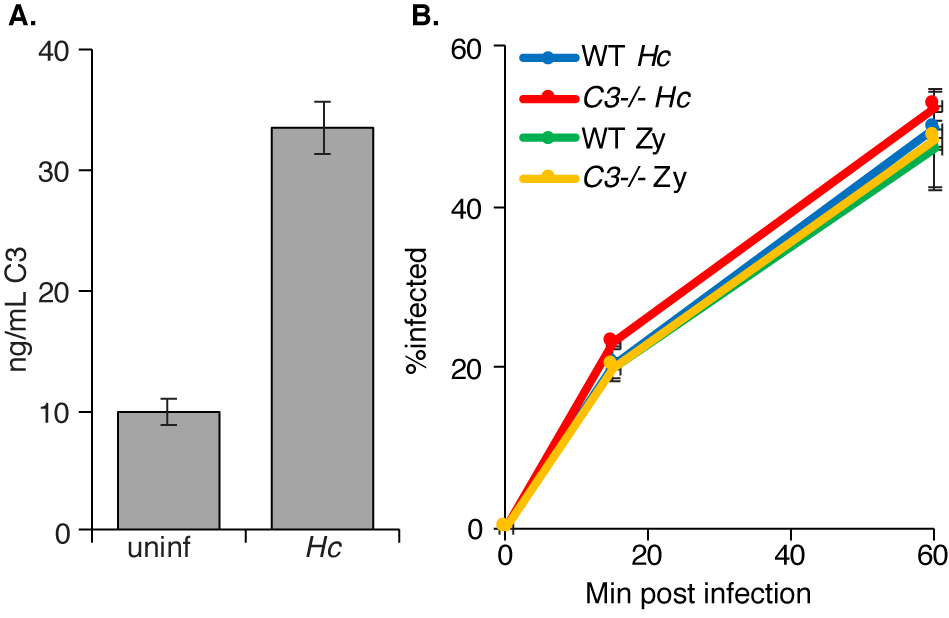

Supplement: S7 Fig — A. BMDMs secrete C3 following infection with Hc. BMDMs were infected with Hc at an MOI2 for 24h, supernatants were harvested and C3 levels were quantified using a BD mouse C3 ELISA kit. B. C3-/- BMDMs are not defective in phagocytosis of Hc yeast or zymosan. WT and C3-/- BMDMs were infected with mCherry-expressing Hc or FITC-labelled zymosan, and uptake over time was measured using flow-cytometry. Extracellular yeasts were identified by Calcofluor White staining. (TIF) [file ppat.1010237.s007.tif]

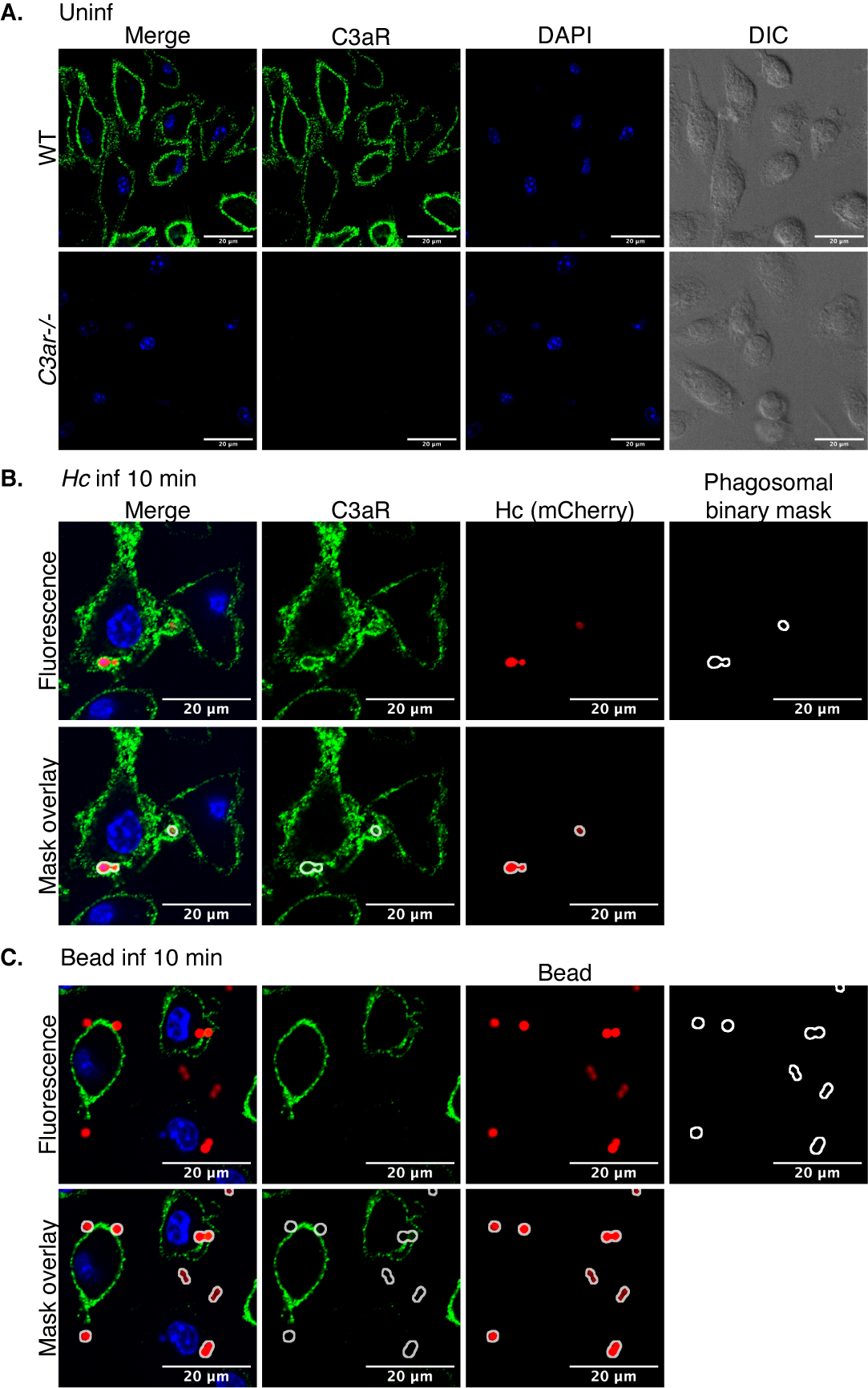

Supplement: S8 Fig — A. Uninfected WT and C3ar-/- BMDMs were stained with a C3aR-specific antibody and imaged using confocal microscopy and optical sectioning. Representative slices of 2 biological replicates are shown. The antibody specifically detects C3aR, as staining was not observed in C3ar-/- BMDMs. C3aR exhibits punctate localization near the plasma membrane in WT BMDMs. B-C. Representative slices from confocal imaging showing the region of interest mask derived from binary operations on Hc (B) or bead (C) fluorescent channel thresholding used to measure the phagosomal C3aR intensity of Hc and bead-containing phagosomes. The original and overlaid fluorescence images are shown. Scale bar = 20 μm. (TIF) [file ppat.1010237.s008.tif]

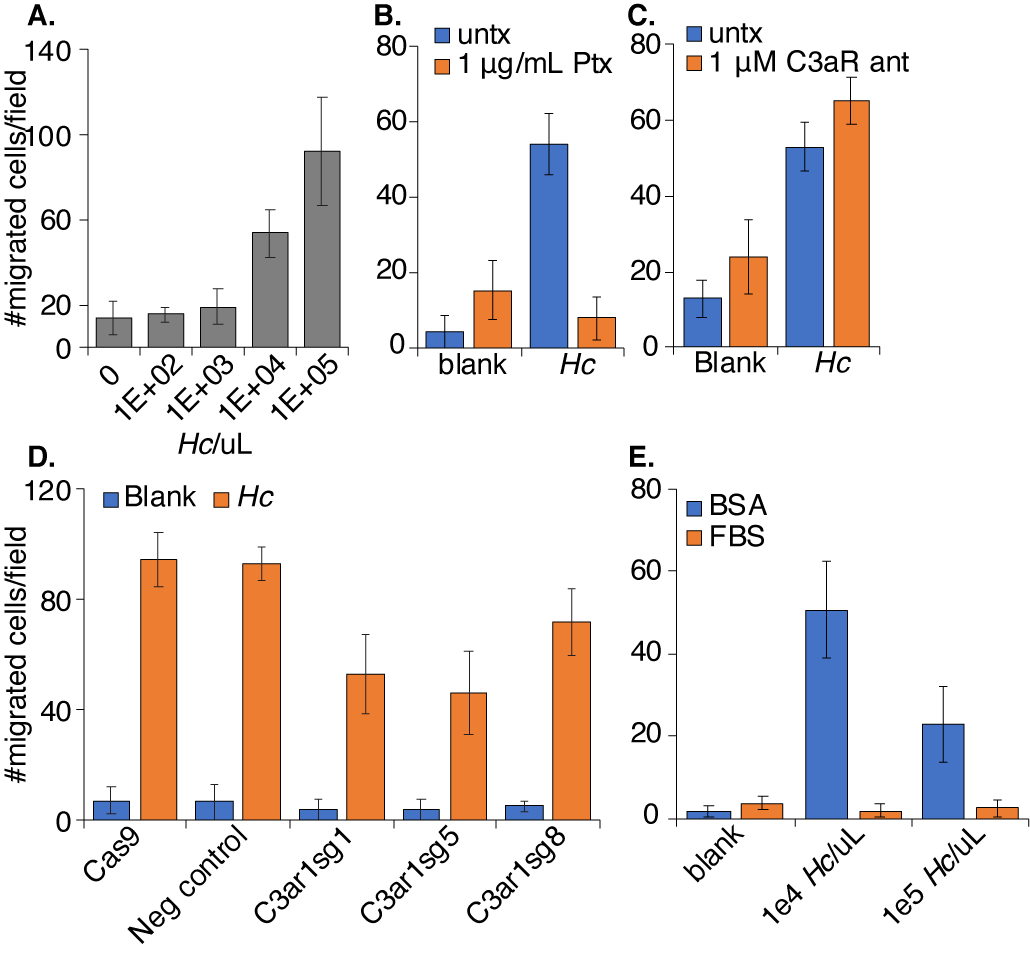

Supplement: S9 Fig — A. Hc stimulates chemotaxis of J774A.1 macrophage-like cells. Hc yeast were seeded into multiple-well plates at varying concentrations, and WT J774A.1 cells were seeded onto transwell permeable supports with 5 μm pores. Serum-free media supplemented with 0.25% BSA was used as the diluent in both the chamber and well unless otherwise indicated. After 3 h of migration, transwells were stained with crystal violet, and non-migratory cells were wiped off of the upper side of the transwell using a Q-tip. The number of migratory cells in each condition was quantified by microscopy (n = 2 biogical replicates, 3 fields/biological replicate). B. Migration towards Hc is Gai-dependent. J774A.1 cells with or without pre-treatment with 1 μg/mL pertussis toxin (PTX) for 2 h were seeded into transwell permeable supports and migration towards 1e5 Hc/uL was quantified as described above. The number of migrating cells was quantified as described. C. The C3aR antagonist does not inhibit macrophage migration towards Hc. J774A.1 macrophages were treated with 1 μM SB290157, a C3aR antagonist, and migration towards Hc was assessed as described. D. C3aR-deficiency moderately impacts migration of J774A.1 cells towards Hc. Cas9-expressing J774A.1 macrophages transduced with non-targeting or C3aR-targeting sgRNAs were assessed for their ability to migrate towards Hc as described previously. E. Hc-dependent migration is abolished in the presence of FBS. The transwell migration assay was performed with media supplemented with BSA or 10% FBS to determine whether FBS affected the migration of macrophage-like cells towards Hc yeast. (TIF) [file ppat.1010237.s009.tif]

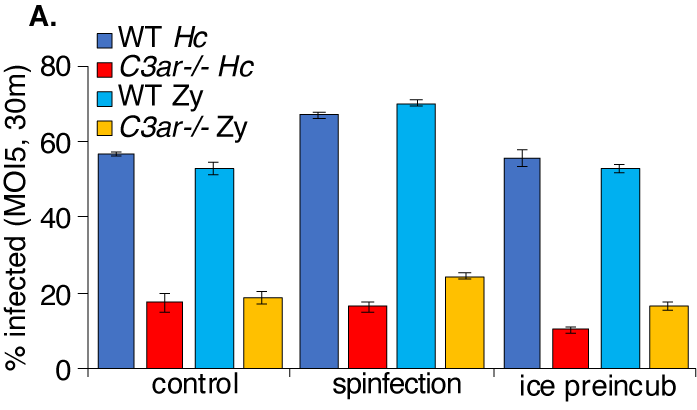

Supplement: S10 Fig — A. BMDMs were infected with Hc or zymosan at an MOI = 5 for 30 min. For the control condition, particles were added to the wells and allowed to settle onto the monolayer without intervention. For the 5 min spinfection, particles were added to the cells, and the plate was spun for 5 min at 550XG at RT before transferring to a 37°C, 5% CO2 incubator. For the ice preincubation condition, BMDMs were pre-chilled for 20 min on ice, and particles were allowed to settle onto the monolayer for 1 h on ice, then were transferred to a tissue culture incubator. Phagocytosis was measured as described previously (n = 3 biological replicates). (TIF) [file ppat.1010237.s010.tif]

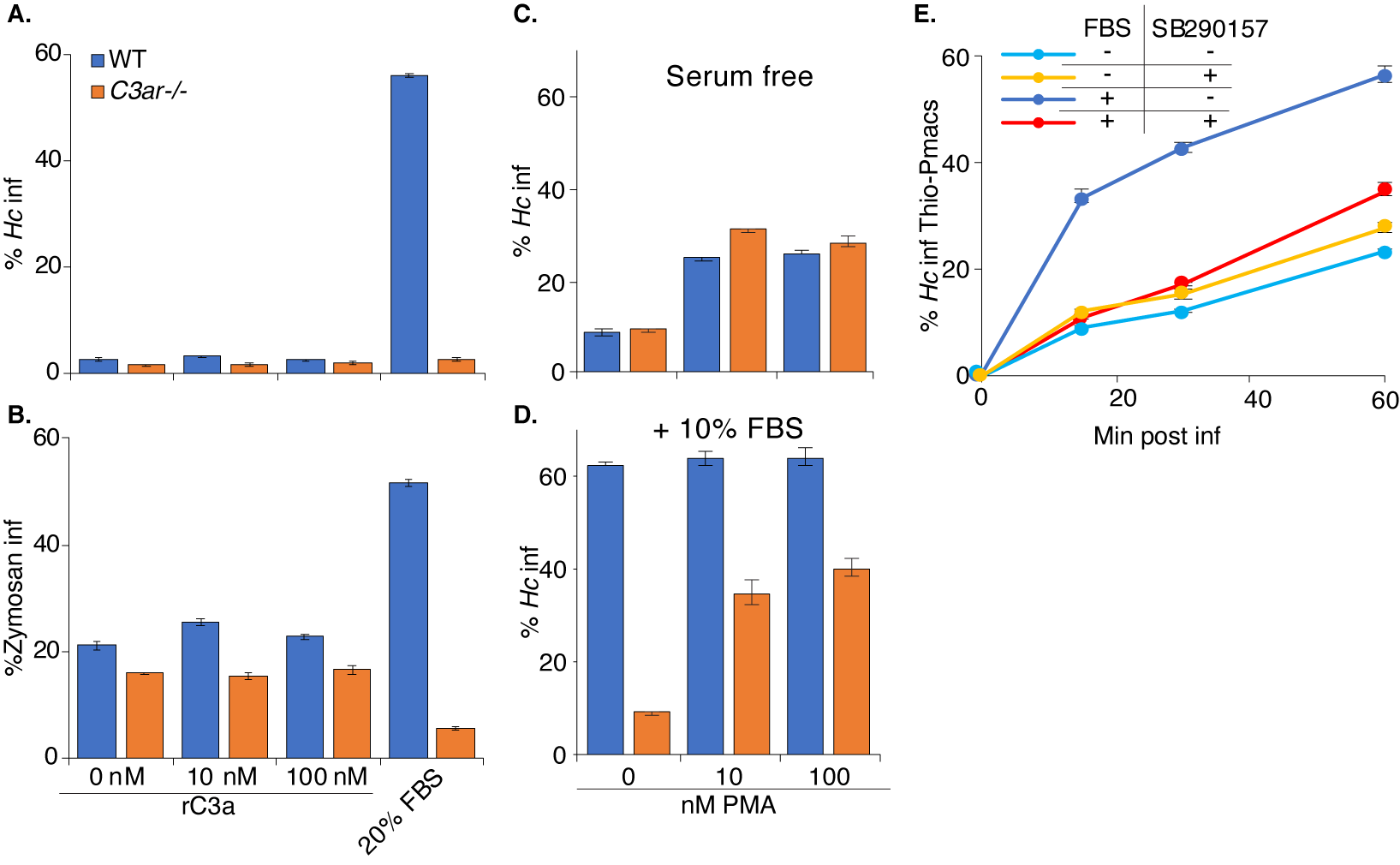

Supplement: S11 Fig — BMDMs were pre-incubated in serum-free media with varying concentrations of recombinant mouse C3a (R&D systems) or with 20% FBS for 1 h, then infected with A. Hc or B. Zymosan at an MOI of 5 for 30 min. Phagocytosis was assessed by flow cytometry (n = 2 biological replicates). C-D. BMDMs In serum-free (C) or FBS-supplemented (D) media were pre-treated with PMA for 2h and infected with Hc (MOI5, 60 min). Phagocytosis was measured by flow cytometry (n = 3 biological replicates). E. Thioglycollate-elicited peritoneal macrophages in serum-free or FBS-supplemented media were treated for 5 min with a C3aR antagonist (SB290157, 10 μM) and infected with Hc. Phagocytosis was assessed by flow cytometry (n = 3 biological replicates). (TIF) [file ppat.1010237.s011.tif]

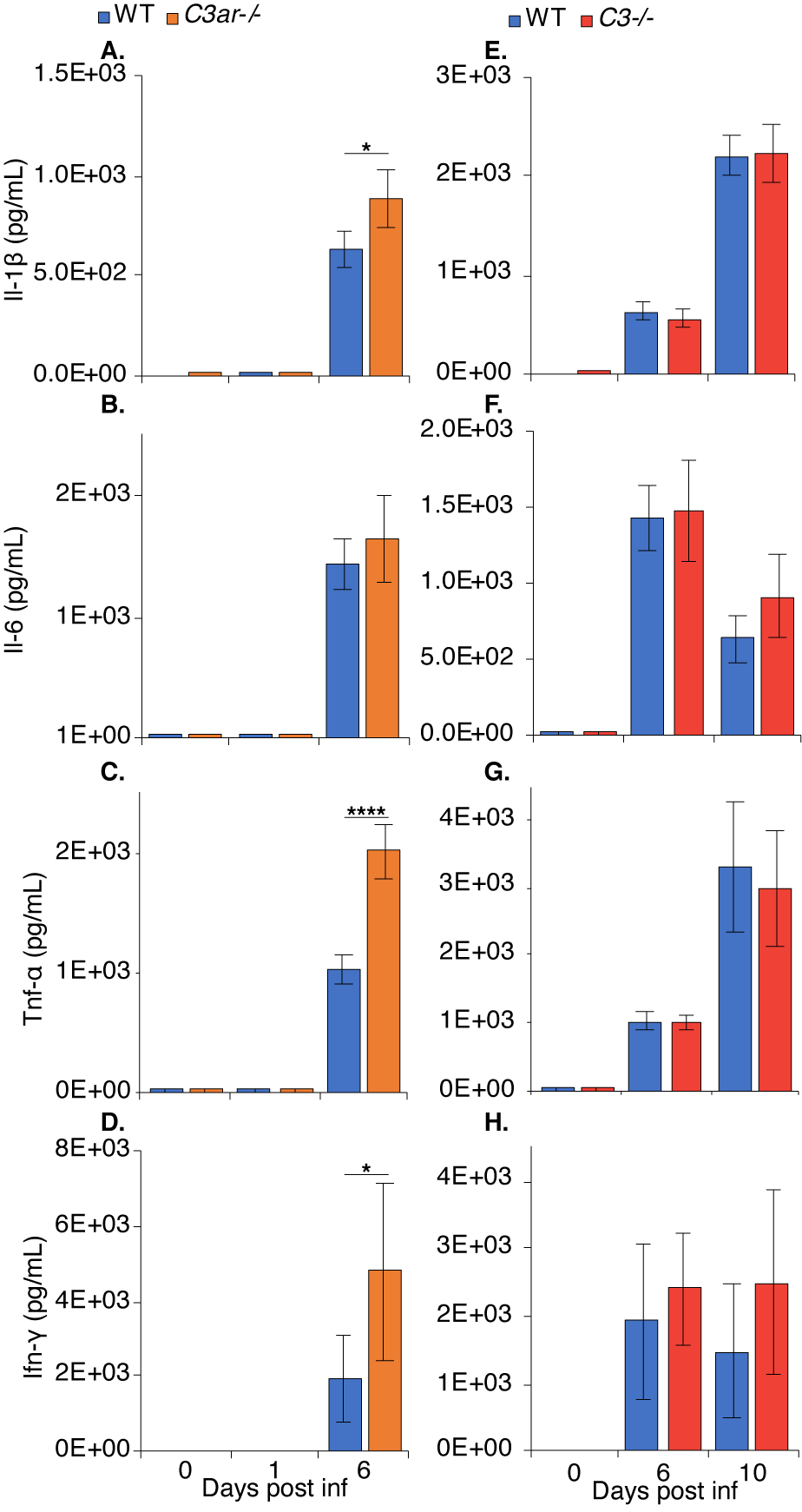

Supplement: S12 Fig — A-D: C3ar-/- (A-D) or C3-/- (E-H) and age-matched WT mice were infected intranasally with Hc. The levels of Il-1β (A,E), Il-6 (B,F), TNF-α (C,G), and IFN-γ (D,H) in lung homogenates were measured at the indicated time points by the cytokine CBA assay. *p ≤ 0.05, ****p ≤ 0.0001, by two-tailed student’s T-test. (TIF) [file ppat.1010237.s012.tif]
